# Hip aspiration culture: analysing data from a single operator series investigating periprosthetic joint infection

**DOI:** 10.5194/jbji-6-165-2021

**Published:** 2021-05-10

**Authors:** Connor J. Barker, Alan Marriot, Munir Khan, Tamsin Oswald, Samuel J. Tingle, Paul F. Partington, Ian Carluke, Mike R. Reed

**Affiliations:** 1 South Tees NHS Foundation Trust, Middlesbrough, TS4 3BW, UK; 2 Northumbria Healthcare NHS Foundation Trust, Cramlington, NE23 6NZ, UK; 3 Northumbria Healthcare NHS Foundation trust, Cramlington, NE23 6NZ, UK; 4 Newcastle upon Tyne Hospitals NHS Foundation Trust, Freeman Hospital, Newcastle upon Tyne, NE7 7DN, UK

## Abstract

**Introduction**: We undertook this study to know the sensitivity, specificity and post-test probabilities of hip aspiration when diagnosing periprosthetic hip infections. We also examined “dry tap” (injection with saline and aspiration) results and aspiration volumes. **Methods**: This is a retrospective cohort study of patients aspirated for suspected periprosthetic joint infection between July 2012 and October 2016. All aspirations were carried out by one trained surgical care practitioner (SCP). All aspirations followed an aseptic technique and fluoroscopic guidance. Aspiration was compared to tissue biopsy taken at revision. Aspiration volumes were analysed for comparison. **Results**: Between January 2012 and September 2016, 461 hip aspirations were performed by our SCP. Of these 125 progressed to revision. We calculated sensitivity 59 % (confidence interval (CI) 35 %–82 %) and specificity 94 % (CI 89 %–98 %). Pre-test probability for our cohort was 0.14. Positive post-test probability was 0.59 and negative post-test probability 0.06. Aspiration volume for infected (n=17) and non-infected (n=108) joints was compared and showed no significant difference. Dry taps were experienced five times; in each instance the dry tap agreed with the biopsy result. **Conclusions**: Our data show that hip aspiration culture is a highly specific investigation for diagnosing infection but that it is not sensitive. Aspiration volume showed no significant difference between infected and non-infected groups. Each time a joint was infiltrated with saline to achieve a result, the result matched tissue sampling.

## 1 Introduction

Periprosthetic joint infection (PJI) is a painful condition that results in early failure of arthroplasty and significant cost (Peel et al., 2013; Cross et al., 2014). Infection burden for all hip replacements in England is reported to be 1.6 % when there is high-quality post-discharge surveillance (Tanner et al., 2013). There is no one gold standard test for PJI: the difficulty in defining it has been much discussed (Parvizi et al., 2011, 2013) and has led to the development of multi-test diagnostic criteria (Parvizi et al., 2018). Diagnoses are currently informed by several factors such as the clinical picture, pre-operative indicators such as blood tests (C-reactive protein, CRP, and erythrocyte sedimentation rate, ESR) and synovial fluid tests (Parvizi et al., 2018). CRP and ESR are sensitive but not specific (Piper et al., 2010), whereas synovial fluid aspiration is an invasive test with operator-dependent success rates (Tingle et al., 2016). In our institution the majority of hip aspirations for suspected PJI are carried out by a single trained orthopaedic surgical care practitioner (SCP). This approach has been proven to lower the rate of dry tap aspirations (Tingle et al., 2016). When unable to obtain a synovial aspirate, the joint was injected with saline and re-aspirated immediately. This method of injecting with saline is not often reported in the literature (Partridge et al., 2018). The primary aim of this study is to assess the utility of joint aspiration from the viewpoint of an accurate and reliable dataset provided by a single operator. This will be done by analysing aspiration culture post-test probabilities, sensitivity and specificity, which are then compared to common serological markers. Secondary measures include comparing aspirate volumes of PJI versus aseptic joints, comparing the use of blood culture bottles versus universal containers and the results of dry tap aspirations.

## 2 Methods

We have analysed all hip aspirations performed for suspected PJI by our SCP between July 2012 and October 2016. In this period, 461 aspirations were performed, of which 107 patients progressed to revision. Patients were included in the study if they had a hip aspiration for suspected PJI and had progressed to revision, and patients were excluded if they had not progressed to revision. The cohort of 107 patients experienced 125 of the 461 aspirations. The larger number of aspirations than patients can be accounted for by 14 repeat aspirations and 4 bilateral aspirations. Of the 125 aspirations, 60 were performed on males and 65 on females, and mean age at time of aspiration was 66 (38–94) and 73 (54–98) respectively. All aspirations were fluoroscopy guided and followed an aseptic technique, including skin preparation with a 2 % chlorhexidine wash. The number of patients receiving antibiotics at the time of aspiration is unknown. All aspirations were performed in a surgical theatre; a theatre with laminar airflow was sometimes but not always available. If an aspiration was dry, a fluid washout was performed by injecting into the joint 10 mL normal saline and immediately re-aspirating (n=5). Contrast media were not used to confirm the source of the aspirate. Specimens were put into an anaerobic blood culture bottle, an aerobic culture bottle and a sterile universal container, all of which were manufactured by Becton Dickinson. At revision, five different tissue specimens were taken with separate sterile instruments (Atkins et al., 1998). Prior to procedure CRP and ESR were obtained in 117 of 125 cases.

### Microbiology

UK protocols for investigation of orthopaedic-implant-associated infections were followed as a minimum standard throughout (Public Health England, 2016). Blood culture bottles were incubated for 10 d and plated out if identified as positive. Where the clinical picture is consistent with PJI but no growth after 10 d, broth cultures were incubated for 14 d as per national procedure (Public Health England, 2016). Aspirates in sterile universal containers are plated out and plates read at either 48 or 72 h, and then an anaerobic plate is read at 5 d. Five drops are put in enrichment broth and plated out when cloudy or at 5 d; these plates are then read at 48 h. If a fungal infection is suspected, a Sabouraud agar plate was used. Tissue was homogenised in 1 mL of sterile saline by glass beads and applied to a vortex for 15 s. The following media were inoculated with each specimen: Robertson's cooked meat broth, chocolate blood agar, blood agar, fastidious anaerobe agar (FAA) + 5 µg metronidazole (one cultivated for 3 d and another for 5 d), and chromogenic UTI media. A single specimen from the entire series was sent for tuberculosis culture. Biopsy results were considered to be infected if three or more of the samples grew the same organism; this was our control test. If two or fewer samples grew the same organism, the result was considered to be not infected. This is an approach proven to be most likely to predict infection from biopsy (Atkins et al., 1998). By definition there were no false positives or false negatives in the tissue sample results. Cut-offs for CRP and ESR were >10.3 mg/L and >13 mm/h respectively, as recommended by meta-analysis (Piper et al., 2010).

When calculating confidence intervals (CIs), an alpha level of 0.05 was used to calculate 95 % CI. Analysis of aspiration volume data was performed using RStudio version 1.1.383 (RStudio, 2015). Aspirations from patients with an infection (confirmed by biopsy) were compared to those confirmed non-infected. A Shapiro–Wilk normality test was performed, followed by a Mann–Whitney test to compare the distribution of aspiration volume for infected and non-infected patients.

## 3 Results

### 3.1 Aspiration culture

The results of our statistical analysis are presented in Table 1; 125 aspiration cultures with subsequent tissue samples were available for analysis. For our population of patients aspirated for suspected PJI, we calculated a pre-test probability of infection to be 0.14. Aspiration culture positive post-test probability and negative post-test probability were 0.59 and 0.06 respectively. At time of tissue sampling, the number of procedures where the patient was on antibiotics at the time of revision was 13; the outcomes of those 13 are presented in Table 2. Pathogens and contaminants, as determined by tissue sample (Atkins et al., 1998), are reported in Table 3. Fourteen results were available to compare the accuracy of blood culture and universal containers, the results of which are displayed in Table 4. Positive post-test probabilities of CRP (>5 mg/L), CRP (>10.3 mg/L) and ESR (>13 mm/h) were 0.21, 0.23 and 0.15 respectively. The negative post-test probabilities of CRP (<5 mg/L), CRP (<10.3 mg/L) and ESR (<13 mm/h) were 0.07, 0.10 and 0.14 respectively.

**Table 1:** Statistical analysis of CRP, ESR and aspiration; biopsy included for reference.

	CRP >5 mg/L	CRP >10.3 mg/L	ESR >13 mm/h	Aspiration	Biopsy
	(n=117)	(n=117)	(n=117)	(n=125)	(n=125)
True positive	13	9	12	10	17
True negative	31	70	31	101	108
False positive	69	30	69	7	0
False negative	5	8	5	7	0
Sensitivity (%) (95 % CI)	76 (56–97)	53 (29–76)	71 (49–92)	59 (35–82)	
Specificity (%) (95 % CI)	51 (41–61)	70 (61–79)	31 (22–40)	94 (89–98)	
Pre-test probability	0.15	0.15	0.15	0.14	0.14
Positive post-test probability	0.21	0.23	0.15	0.59	
Negative post-test probability	0.07	0.10	0.14	0.06	
Positive predictive value (%) (95 % CI)	21 (11–31)	23 (10–36)	15 (7–23)	59 (35–82)	
Negative predictive value (%) (95 % CI)	93 (86–99)	90 (86–99)	86 (74–97)	94 (89–98)	

**Table 2:** Outcomes of patients receiving antibiotics at the time of revision.

Species	Number
True positive	1
True negative	10
False positive	2
False negative	0

**Table 3:** Pathogens and contaminants. Pathogenic indicates that three or more tissue samples grew concordant species. Contaminants are cases where fewer than three tissue samples grew concordant species; this has been subdivided to show when a single or two tissue samples were culture positive.

Species	Pathogenic instances	Contaminant (1)	Contaminant (2)
*Staphylococcus epidermidis*	7	5	1
*Propionibacterium acnes*	3	4	2
*Staphylococcus aureus*	2		
*Candida* sp.	1		
*Pseudomonas aeruginosa*	1		
*Staphylococcus capitis*	1		
*Staphylococcus lugdenensis*	1		
*Staphyloccus saccharolyticus*	1		
*Streptococcus milleri*			1
*Staphylococcus warneri*			1
*Enterococcus faecalis*		1	
*Coagulase negative Staphylococcus* sp.		1	
*Microccus luteus*		1	
*Staphylococcus cohnii*		1	
*Staphyloccus cohnii urealyticum*		1	

**Table 4:** Comparison of blood cultures with universal containers; 14 of 17 cases positive for PJI were available for comparison. In three cases no universal container was sent for analysis.

	Blood culture bottle	Universal container
True positive	9	5
True negative	1	1
False positive	4	4
False negative	0	4

### 3.2 Aspiration volume

Aspiration volume for infected (n=17) and non-infected (n=108) joints was compared. The mean volume for infected and non-infected joints was 6 mL (2–36 mL) and 11 mL (1–200 mL) respectively. We ascertained that both infected and non-infected groups did not display a normal distribution (W=0.5573, p<0.001 and W=0.39955, p<0.001 respectively). Hence, comparison of the two populations required a non-parametric test, which could not disprove that they did not come from the same distribution (W=758, p=0.2458).

### 3.3 Dry tap

The five aspirations that were dry taps had 10 mL of normal saline infiltrated and then immediately aspirated. Two grew an organism (*P. aeruginosa*, *S. aureus*), whereas three did not. Compared to tissue sampling, *P. aeruginosa* was cultured in three of five samples, and *S. aureus* was cultured in five of five samples. Of the three aspirates which were sterile, two had no positive tissue cultures and one was culture positive for *P. acnes* in one of five samples. According to our definition, all five aspirations agreed with the biopsy result.

## 4 Discussion

PJI diagnostics is a difficult clinical problem in which a range of diagnostic tests inform decision making. Our data suggest that a positive synovial fluid culture may well be erroneous (positive post-test probability 0.59) but that a negative sample is highly likely to be negative (negative post-test probability 0.06). In our population the pre-test probability of infection was 0.14. These data suggest the following for our cohort: before an aspiration is performed, 14 out of 100 individuals truly have a PJI; after a positive aspirate, 59 in 100 individuals truly have a PJI and 41 in 100 do not. After a negative aspirate 6 in 100 do have an infection, whereas 94 in 100 individuals do not.

A recent review of serum biomarkers in PJI reported the sensitivity of CRP and ESR to range from 74 % to 94 % and from 42 % to 94 % respectively; specificity of CRP and ESR ranged from 20 % to 100 % and from 33 % to 87 % respectively (Saleh et al., 2018). The wide range of values above belies the difficulty in studying serological markers when assessing PJI. Our data are mostly in keeping with the previous literature, though in this cohort of patients the specificity of ESR was lower than other similar studies (Saleh et al., 2018).

We decided to compare the threshold for CRP from a meta-analysis (Piper et al., 2010) versus the common clinical value of >5 mg/L. Our data show that <5 mg/L would be a more reliable value to rule out infection in our cohort. The data from our cohort indicate that >5 mg/L is comparable to >10.3 mg/L when positively identifying infection. We would state that neither CRP nor ESR are suitable as positive identifiers of infection, though CRP <5 mg/L has a strong negative predictive value. A conclusion from our results is that CRP is a more useful blood marker than ESR as a sole indicator.

A recent large retrospective analysis offered that sensitivity of a dry tap is comparable to a normal aspiration, whereas specificity was lower (Partridge et al., 2018). This is in keeping with previous findings in a much smaller cohort (Ali et al., 2006). Of our five dry taps we determined that aspiration culture was in agreement with the tissue culture in each instance. One patient grew *P. acnes* in a single tissue sample; in that instance the patient's CRP was 1 mg/L and ESR 8 mm/h, suggesting that the single tissue sample was a contaminant. We would encourage future authors to report data on dry taps to increase the body of evidence.

We were unable to discern a difference in aspiration volume between the infected and non-infected groups. Fluid volume difference was found to be not significant in a previous study (Ali et al., 2006), though again the cohort was small, and so we would encourage authors to report these data. Synovial fluid was collected in an aerobic, anaerobic and sterile universal container. We hypothesised that the sterile containers were less accurate, as reported in a previous study (Jordan et al., 2014). The appearance that universal containers are less accurate is in keeping with previous opinion within the laboratory. In the future our lab does not plan to use universal containers when aspirating a hip joint for periprosthetic joint infection.

Limitations of this study include not considering patient factors such as age, gender, disease state, medication state or type of implant. There is a methodological limitation in some, but not all specimens were cultured for 14 d, which may lower detection of slow-growing bacteria such as *P. acnes* (Schwotzer et al., 2014); given *P. acnes* was the most common pathogen and contaminant in this study, this may be a point for further study. Setting tissue sampling as a control test is recognised as a limitation of the study due to the risk of culture-negative infections, a recognised phenomenon (Tan et al., 2018).

In this study we used thresholds for CRP derived from a meta-analysis (Piper et al., 2010) and common clinical practice. If we were looking for a rule-out test, then CRP at a threshold of 5 mg/L is most useful. This competes very effectively with aspiration culture, although aspiration has other opportunities for analysis, including white cell count and differential, alphadefensin, and synovial CRP, DNA analysis and others (Dinneen et al., 2013; Bingham et al., 2014; Bonanzinga et al., 2017; Gollwitzer et al., 2013; Gallo et al., 2018; Tetreault et al., 2014; Gomez et al., 2012). Given the increased treatment burden of aspiration, the authors only recommend its use in association with other synovial tests.

## Data Availability

The data used for this article are available on reasonable request.

## Code availability

The code used for analysing aspiration volume distributions is available on request.
